# Supplementary figures and images for: MYB transcription factors in alfalfa (Medicago sativa): genome-wide identification and expression analysis under abiotic stresses
Source: PeerJ. 2019 Sep 17;7:e7714. doi: 10.7717/peerj.7714 (PMC6753925; doi:10.7717/peerj.7714)

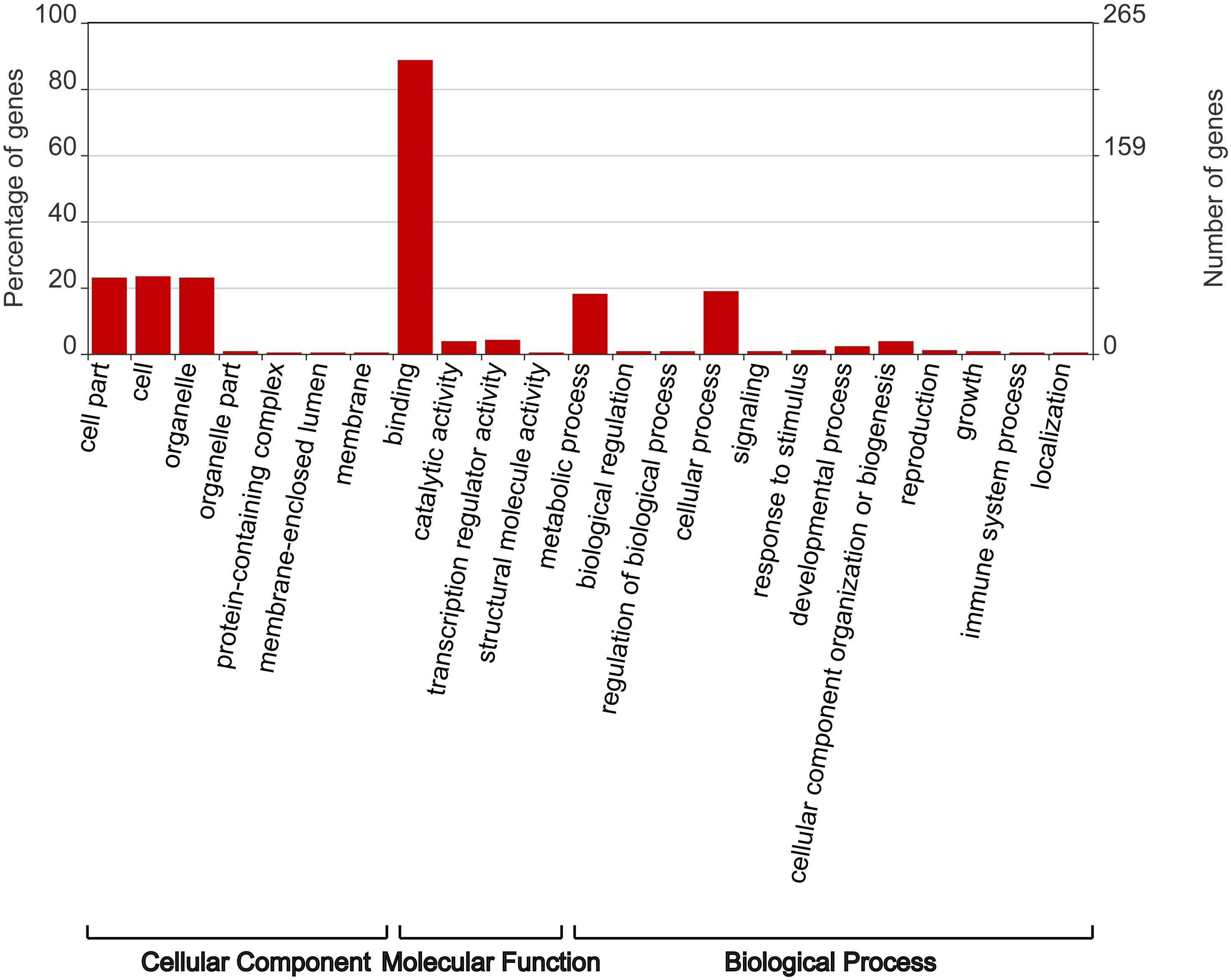

Supplement: Supplemental Information 1 [file peerj-07-7714-s001.png]

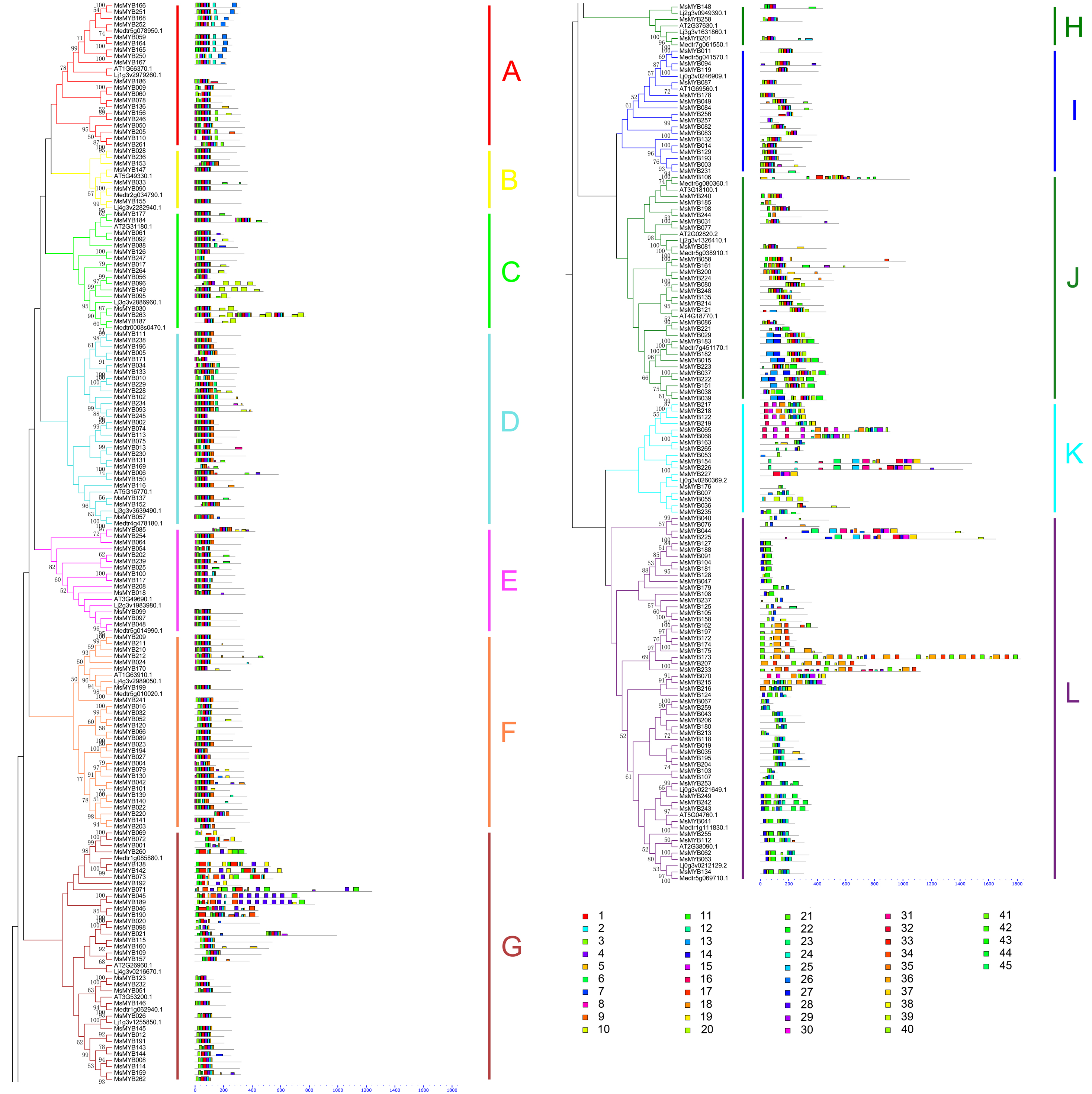

Supplement: Supplemental Information 2 — Motifs are indicated by different colors. The order of the motifs corresponds to the position of the motifs in individual protein sequences. [file peerj-07-7714-s002.png]

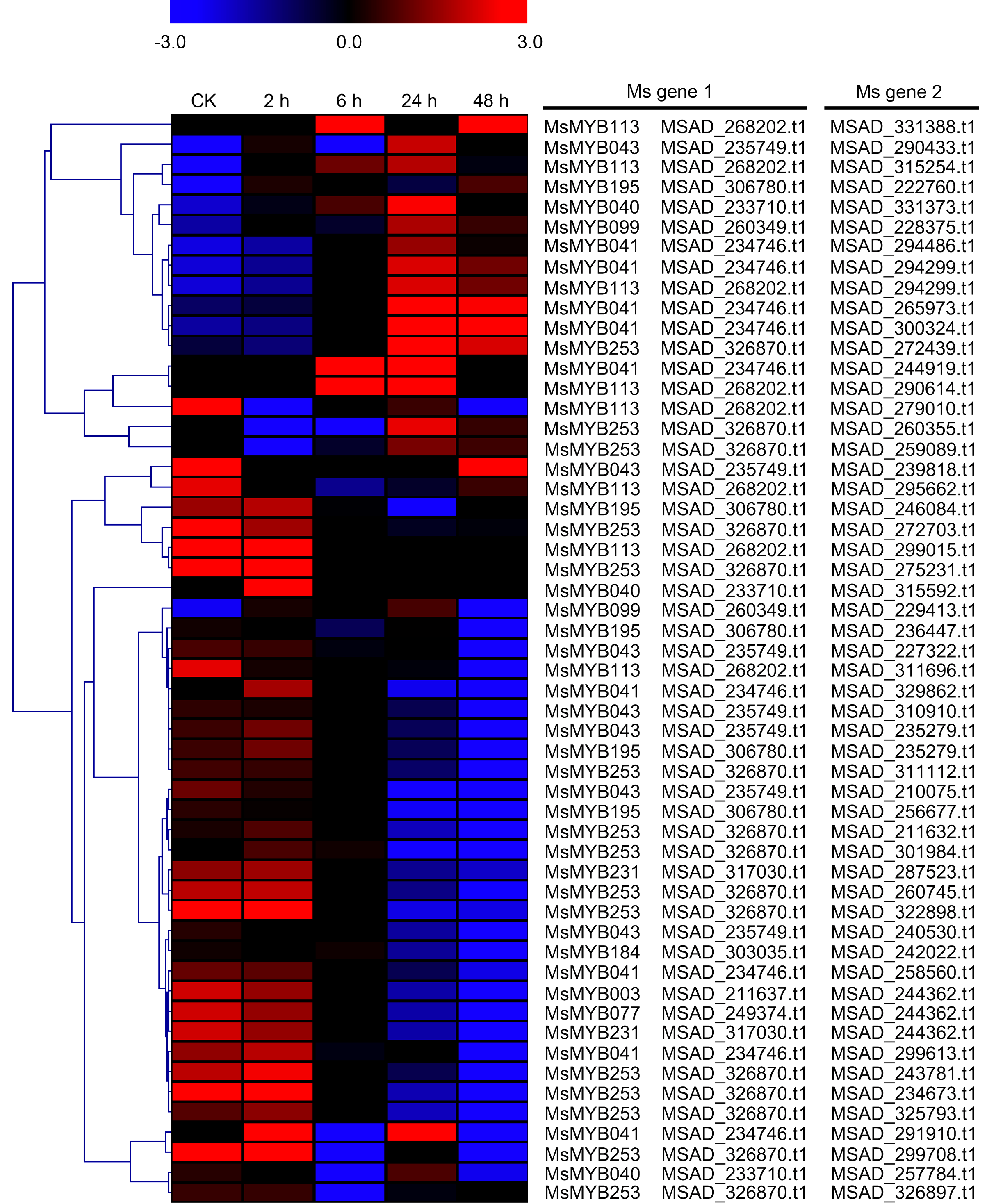

Supplement: Supplemental Information 3 — These interaction genes were predicted by protein-DNA interaction, and “CK” indicates 0 h. The expression levels of these interaction genes with the absolute value of fold change ≥ 10 and the correlation coefficient between MsMYB genes and its interaction genes was more than 0.8. Microarray data were obtained from the reported study in alfalfa (Zhou et al., 2018). [file peerj-07-7714-s003.png]

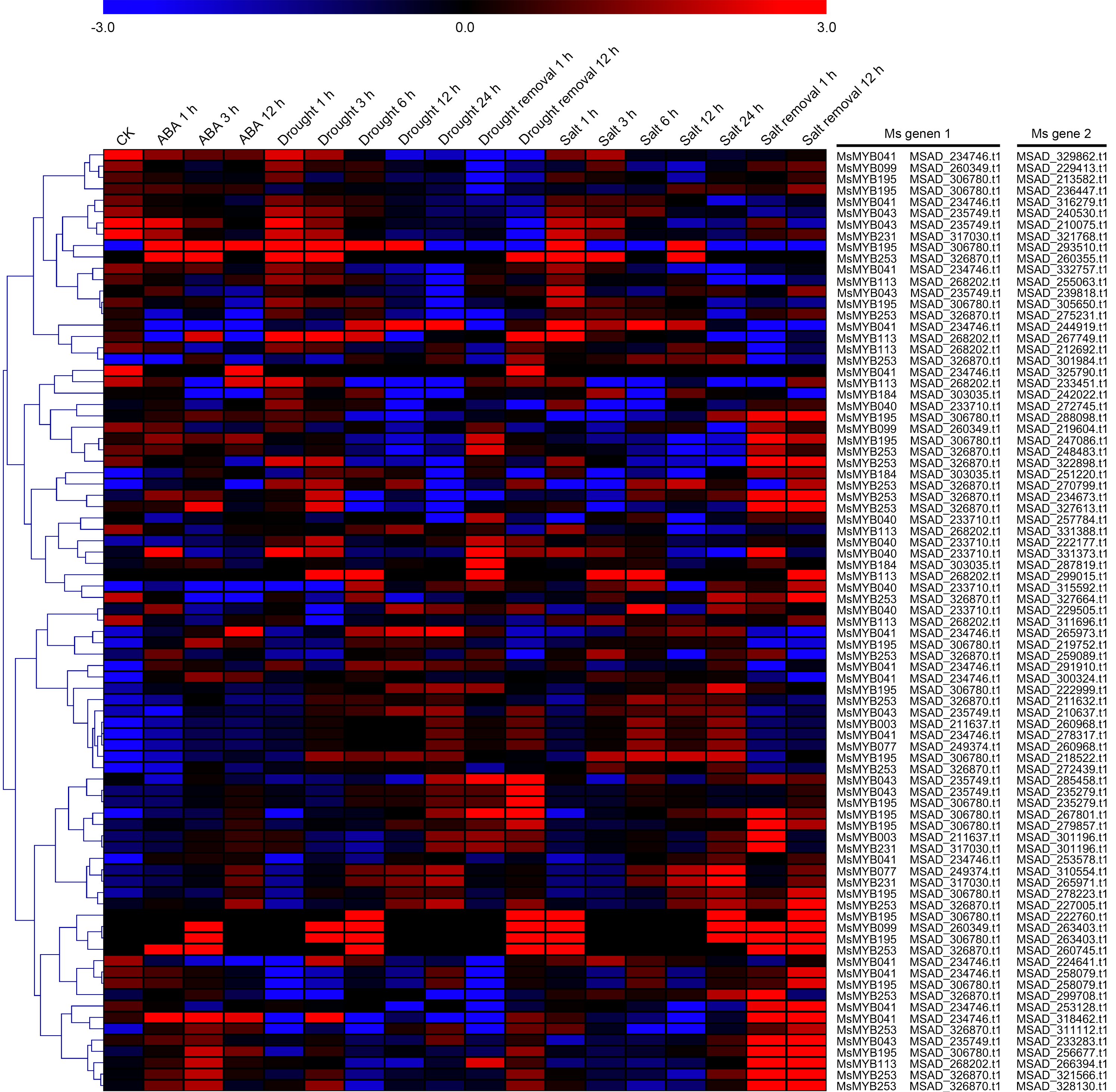

Supplement: Supplemental Information 4 — These interaction genes were predicted by protein-DNA interaction, and “CK” indicates 0 h. The expression levels of these interaction genes with the absolute value of fold change ≥ 10 and the correlation coefficient between MsMYB genes and its interaction genes was more than 0.8. Microarray data were obtained from the reported studies in alfalfa (Luo et al., 2019a, 2019b). [file peerj-07-7714-s004.png]

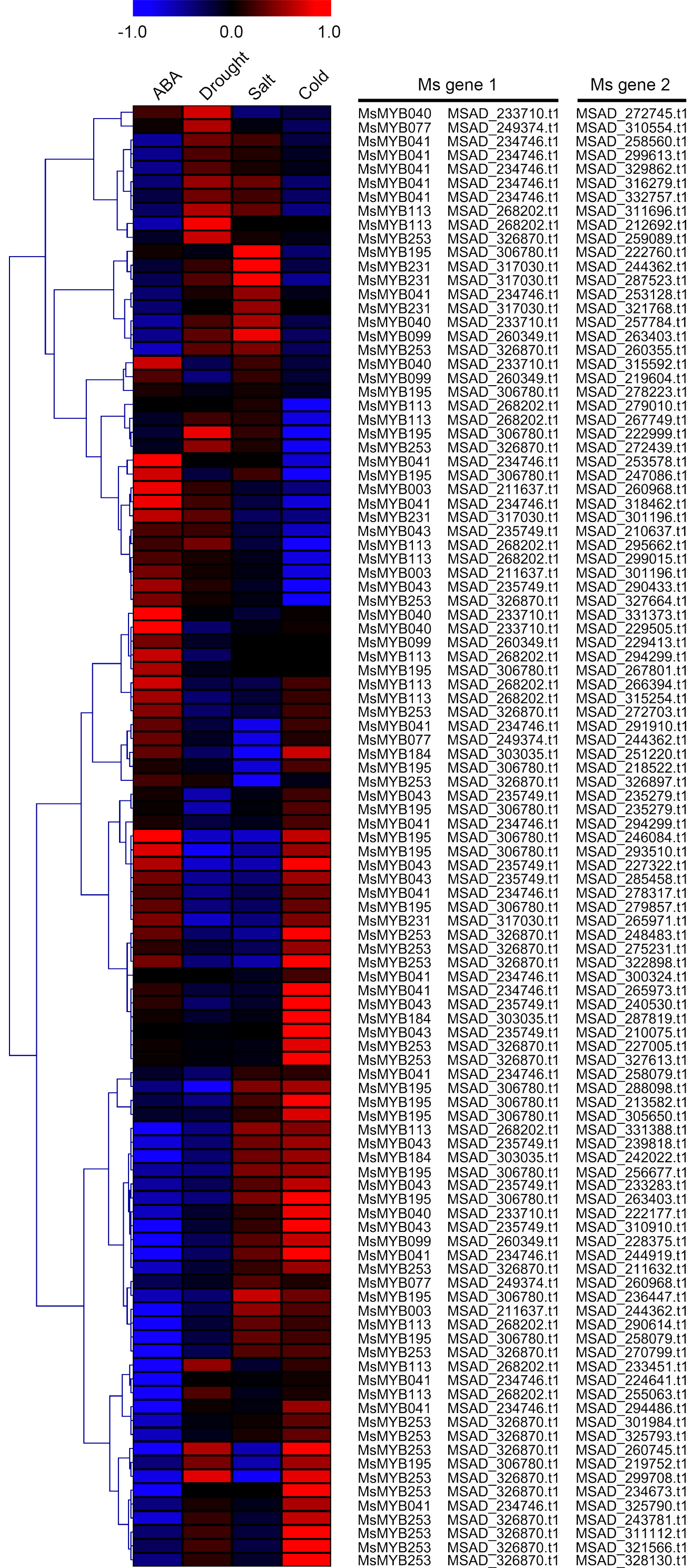

Supplement: Supplemental Information 5 — These interaction genes were predicted by protein-DNA interaction. The expression levels of these interaction genes with the absolute value of fold change ≥ 10 and the correlation coefficient between MsMYB genes and its interaction genes was more than 0.8. [file peerj-07-7714-s005.png]

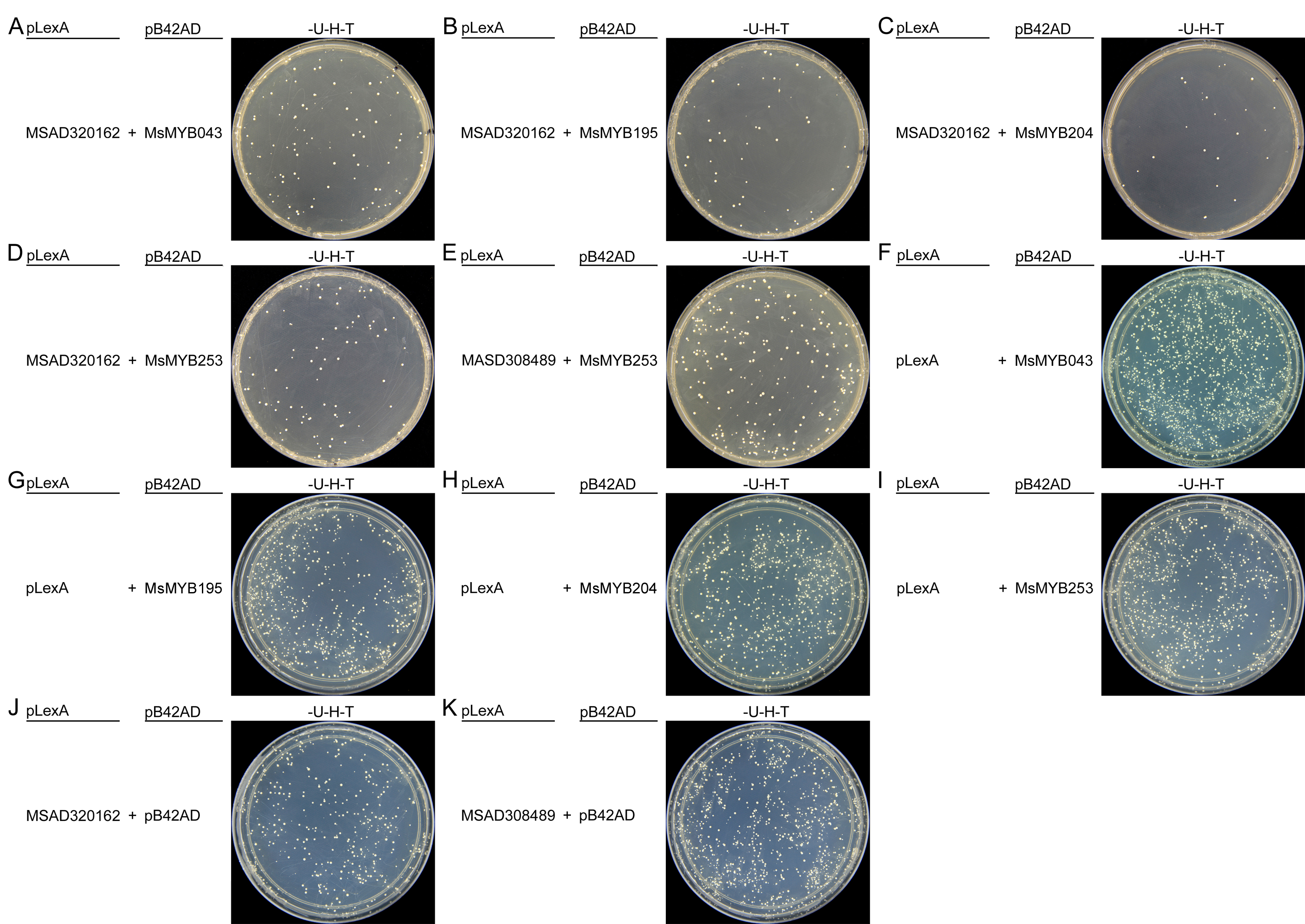

Supplement: Supplemental Information 6 [file peerj-07-7714-s006.png]

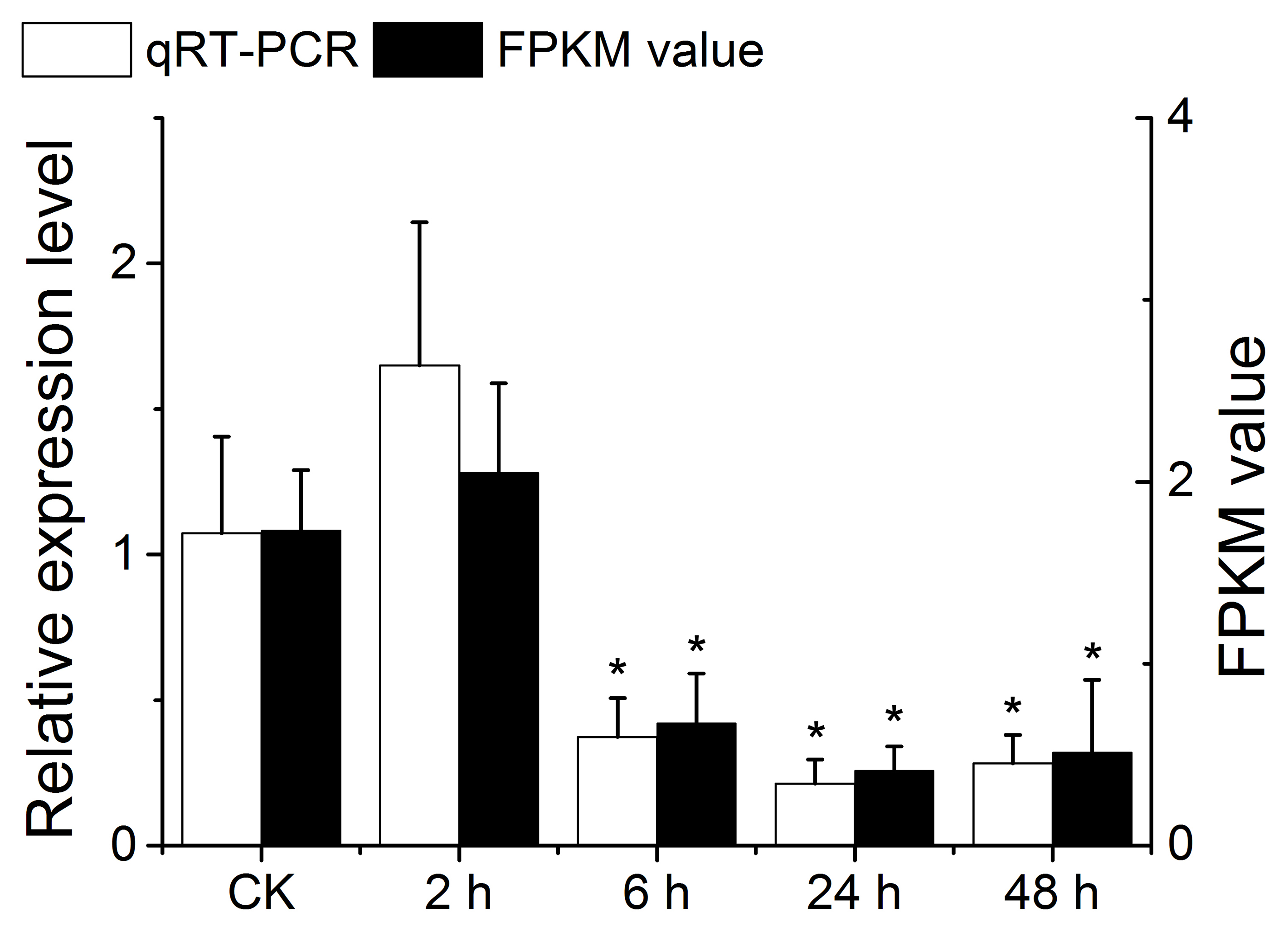

Supplement: Supplemental Information 7 — White bars represent the relative expression levels determined by qRT-PCR (left y-axis). Black bars indicate the transcript abundance change based on the Fragments Per Kilobase per Million Fragments mapped (FPKM) values of the RNA-Seq analysis (right y-axis). Error bars indicate standard errors of the means (n = 3). “*” indicate significance at the 0.05 level. [file peerj-07-7714-s007.png]
